# Potential therapeutic mechanism of genistein in breast cancer involves inhibition of cell cycle regulation

**DOI:** 10.3892/mmr.2014.2907

**Published:** 2014-11-10

**Authors:** LING ZHANG, BO YANG, KE ZHOU, HUA LI, DONG LI, HUI GAO, TAO ZHANG, DONG WEI, ZHIHUI LI, YONG DIAO

**Affiliations:** 1 Department of Radiotherapy, Chengdu Military General Hospital, Chengdu, Sichuan 610083, P.R. China; 2 Department of Oncology, Chengdu Military General Hospital, Chengdu, Sichuan 610083, P.R. China; 3 PET-CT Center, Chengdu Military General Hospital, Chengdu, Sichuan 610083, P.R. China

**Keywords:** genistein, breast cancer, differentially expressed genes, cell cycle

## Abstract

Genistein can prevent tumorigenesis and reduce the incidence of diseases that are dependent upon estrogen. Previous research, however, has shown that genistein can also increase the risk of breast cancer. Thus, the aim of the present study was to investigate the mechanism underlying the effect of genistein in breast cancer and to determine whether genistein produces a therapeutic effect or promotes the development of breast cancer. Gene microarray data obtained from three samples treated with alcohol (control group), three samples treated with 3 μmol/l genistein and three samples treated with 10 μmol/l genistein for 48 h, were downloaded from the Gene Expression Omnibus database. Analysis of the differentially expressed genes (DEGs) and functional enrichment in the two genistein groups was performed. The interaction networks of the DEGs were constructed and the overlapping network was extracted. Finally, the functions and pathways of the DEGs in the overlapping network were enriched. In total, 224 DEGs coexisted in the two genistein groups, and the most significant function of these was the cell cycle. The number and the fold change of expression values of the DEGs in the 10 μmol/l genistein group were significantly higher compared with that of the 3 μmol/l genistein group. The most significant function and pathway of the DEGs in the overlapping network was the cell cycle involving several genes, including GLIPR1, CDC20, BUB1, MCM2 and CCNB1. Thus, genistein stimulation resulted in gene expression changes in breast cancer cell lines and discrepancies increased with higher doses of genistein. The DEGs were most significantly associated with cell cycle regulation.

## Introduction

Breast cancer, is a major public-health issue worldwide, and is the most common type of cancer in females (1). Approximately 25% (2) of all females diagnosed with breast cancer succumb to their disease, despite being treated according to the clinical guidelines (3). The causes of breast cancer have been widely investigated to improve disease prevention and diagnosis. Susceptibility to breast cancer has been attributed to a small number of highly penetrant mutations and a large number of low-penetrant variations (4). The mutations of the tumor suppressor genes breast cancer 1 (BRCA1) and BRCA2, have been demonstrated to be closely associated with breast cancer (5,6). However, the complex pathogenesis remains controversial and is under investigation.

Genistein is the simplest isoflavonoid, which exists extensively in the Leguminosae (7), and is often used as a cancer chemopreventive agent. Previous research has demonstrated that genistein can reduce the incidence of diseases that are dependent upon estrogen, and functions in the prevention of tumors, cardiovascular disease and osteoporosis (8). Furthermore, genistein has been demonstrated to be effective in the prevention of chemically induced mammary tumors in rats (9). This has been attributed to the promotion of cell differentiation and inactivation of the epidermal growth factor signaling pathway (10). Conversely, research has shown that dietary genistein can stimulate mammary gland growth and enhance the growth of MCF-7 cell tumors in ovariectomized athymic mice (11). A ≥10 μmol/l dose of genistein in *in vitro* experiments has confirmed its effectiveness in breast cancer treatment (12). However, dietary treatment with genistein at physiological concentrations produces blood levels of genistein (0.39–3.36 μmol/l) that are sufficient to stimulate estrogenic effects, such as breast tumor growth (13). Therefore the effects of different concentrations and doses of genistein in the prevention or promotion of breast cancer remain unclear.

The present study investigated the potential mechanism underlying the effects of genistein and the influence of different genistein concentrations on breast cancer. Microarray data analysis was used to compare the gene expression profiles of the MCF-7 human breast cancer cell line, treated with 3 and 10 μmol/l genistein, with MCF-7 cells treated with alcohol.

## Materials and methods

### Affymetrix microarray data

The gene microarray data of GSE5200 (14), including three MCF-7 human breast cancer cell samples treated with 0.1% alcohol (control group) for 48 h, three MCF-7 human breast cancer cell samples treated with 3 μmol/l genistein for 48 h and three MCF-7 human breast cancer cell samples treated with 10 μmol/l genistein for 48 h, were downloaded from the Gene Expression Omnibus (GEO) database (http://www.ncbi.nlm.nih.gov/geo/). The Affymetrix Human Genome U133A Array (GPL96) was applied for the analysis of gene expression profiling, and annotation information for all the probe sets was obtained from Affymetrix (Santa Clara, CA, USA).

### Preprocessing of the raw data and differentially expressed gene (DEG) analysis

Data preprocessing and normalization were performed using the Support Vector Regression (15). The raw data of all the samples were converted to an expression profile format. The missing data were then imputed (16), and the complete data were normalized using Support Vector Regression (15). Statistical analysis was performed using the LIMMA (Linear Models for Microarray Data) package in R language (17) to identify the DEGs in the groups treated with 3 μmol/l and 10 μmol/l genistein compared with the control group. The threshold was set at P<0.05 and |logFC| >1.

### Functional enrichment of DEGs

The sequences of the DEGs selected in the 3 and 10 μmol/l genistein groups were mapped using the Clusters of Orthologous Groups (COG) database (http://www.ncbi.nlm.nih.gov/COG) (18) with BLASTX software (19) (similarity threshold, E-value <1E-5), to obtain the functional annotation and COG classification of the DEGs. Through COG classification, the functions of the DEGs in the breast cancer cells treated with different concentrations of genistein, were represented visually and were subsequently analyzed.

### Construction of the interaction network

The combination and dissociation of proteins is required for vital physiological activities and the responses of cells to the external and internal environment are based on the signal transduction networks formed by protein-protein interaction (PPI) networks (20). It is therefore necessary to investigate PPI networks to understand biological processes (21). In the present study, the interaction networks of the DEGs in the two groups treated with genistein were constructed using Osprey software (22), which is designed to enhance the understanding of interaction networks and protein complexes. This software is integrated with the Biomolecular Interaction Network Database (BIND) (23) and Global Resource Information Database (GRID) (23,24), which include >50,000 interactions among protein and nucleotide sequences. The interaction networks of the two groups were integrated and the overlapping network was abstracted for subsequent analysis.

### Functional enrichment analysis of the genes in the overlapping network

Gene set enrichment analysis is based on a group of genes that possess common or relevant functions as compared with the traditional single gene analysis. The variation in biological function is considered to be related to the expression profile of the gene sets rather than individual genes (24,25). In the present study, the DEGs obtained in the overlapping network of the two interaction networks, underwent functional enrichment analysis using the Database for Annotation Visualization and Integrated Discovery (DAVID) (26) software, with a false discovery rate (FDR) <0.05.

### Pathway enrichment of the genes in the overlapping network

The pathway enrichment analysis of the DEGs that were identified in the overlapping network, which was obtained from the two groups treated with 3 and 10 μmol/l genistein respectively, was performed using WebGestalt (27,28) software. The statistical threshold was set to FDR <0.05.

## Results

### Screening of the DEGs

**Figure 1:**
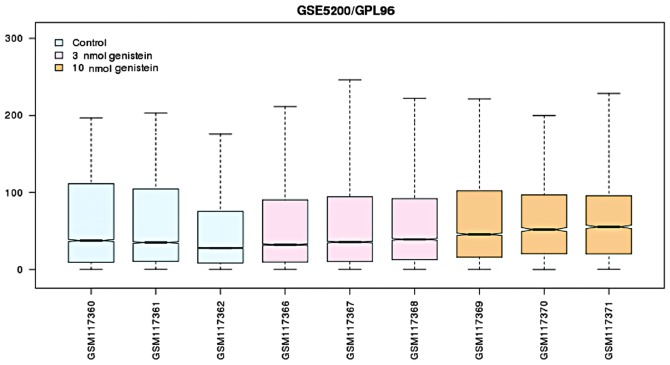
Box-plot of normalized expression data. The control group, 3 and 10 μmol/l genistein groups are represented by blue, pink and orange boxes, respectively. The horizantal black line within the box represents the median, and an equal median indicates an accurate normalization method.

**Figure 2:**
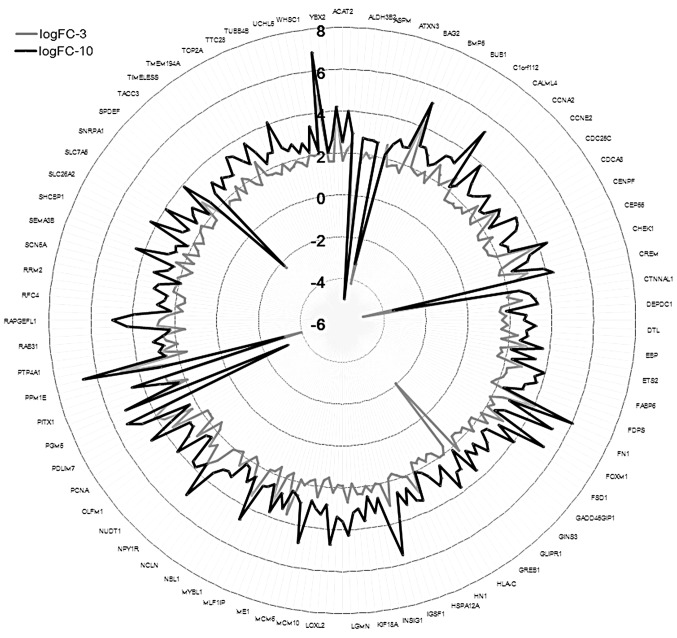
Comparison of the differentially expressed genes identified in the 3 and 10 μmol/l genistein-treated groups.

After preprocessing, the standardized expression profile (Fig. 1) was subjected to differential analysis. The results showed that 544 and 729 DEGs were screened out in the 3 and 10 μmol/l genistein group, respectively (P<0.05 and |logFC| >1). The number of DEGs in the 10 μmol/l genistein group was markedly greater as compared with that of the 3 μmol/l genistein group. Furthermore, there were 224 DEGs that were present in both groups (Fig. 2). The number and the fold change of expression values of the DEGs in the 10 μmol/l genistein group were significantly higher as compared with the 3 μmol/l genistein group.

### Functional enrichment analysis of the DEGs

**Figure 3:**
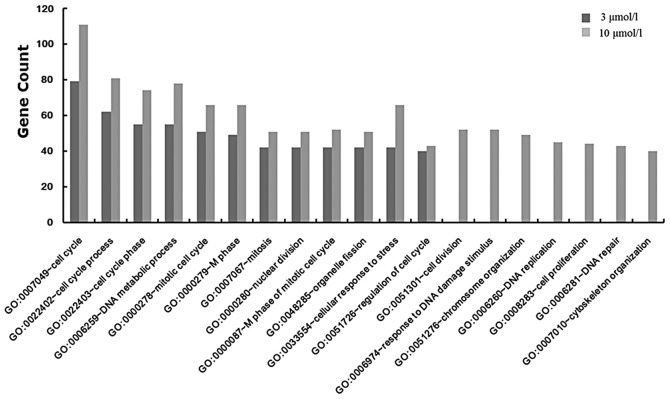
Functional classification of the differentially expressed genes in the two groups treated with 3 μmol/l and 10 μmol/l genistein.

To determine the function of the DEGs in the two groups treated with genistein, the DEGs were mapped to the COG database. Twelve functional nodes were identified in the group treated with 3 μmol/l genistein, and 19 functional nodes were identified in the group treated with 10 μmol/l genistein (Fig. 3). In the 10 μmol/l genistein group, there were seven functional nodes, which included cell division, DNA damage response, chromosome organization, DNA replication, cellular proliferation, DNA repair and cytoskeleton organization; and 12 functional nodes that were shared in both groups. The most significant function of the DEGs in the 3 (FDR=3.29×10^−17^) and 10 μmol/l genistein groups (FDR=4.31×10^−26^) was the cell cycle (GO:0007049).

### Interaction networks of the samples treated with genistein

**Figure 4:**
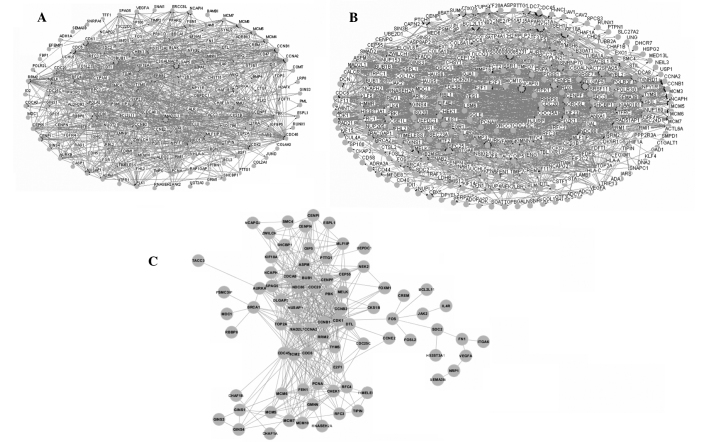
Interaction networks of the differentially expressed genes in the (A) 3 μmol/l and (B) 10 μmol/l genistein group. (C) The overlapping network produced from the two networks.

The interaction networks of the DEGs in the two groups were constructed using Osprey software. The networks of the groups treated with 3 and 10 μmol/l genistein are shown in Fig. 4A and B, respectively. These two networks were merged and the overlapping network was extracted (Fig. 4C). The overlapping network consisted of 49 DEGs and 499 edges.

### Functional enrichment analysis of the genes in the overlapping network

In order to investigate the potential functions of the DEGs, the 49 DEGs were subjected to functional enrichment analysis using DAVID software. The results indicated that the 49 genes clustered into 15 functional terms (Table I), including the cell cycle (FDR=4.02×10^−31^), nuclear division (FDR=1.39×10^−27^) and mitosis (FDR=1.39×10^−27^). The most significant function was the cell cycle (GO:0007049, FDR=4.02×10^−31^), which contained 47 genes, including cell division cycle 20 (CDC20), spindle checkpoint gene (BUB1), mini-chromosome maintenance (MCM) complex 2 and cyclin B1 (CCNB1).

### Pathway enrichment of the genes in the overlapping network

**Figure 5:**
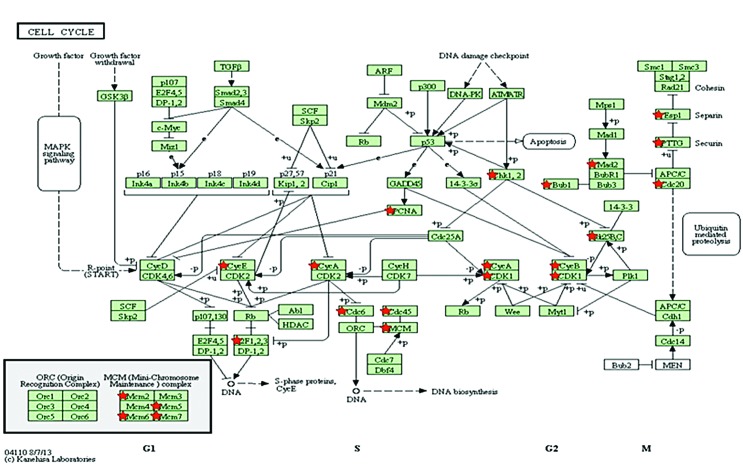
Cell cycle pathway. Red markers represent the differentially expressed genes included in the overlapping network.

In order to understand the pathway and function of the DEGs in the overlapping network, the 49 DEGs underwent pathway enrichment analysis using the WebGestalt software. The results (Table II) indicated that three pathways were significantly enriched, including the cell cycle (FDR=1.52×10^−16^), DNA replication (FDR=5.95×10^−7^) and oocyte meiosis (FDR=3.16×10^−5^). The cell cycle was the most significant pathway, containing 20 DEGs (Fig. 5), including CDC20, MCM2, CCNB1 and BUB1. These data indicated that the DEGs were involved in different phases of the cell cycle.

## Discussion

Breast cancer is the most commonly diagnosed type of cancer among females. Although certain genetic mutations have demonstrated an association with the development of breast cancer, such as p53 and BRCA1 (29,30), there remain numerous unanswered questions regarding the etiology of this disease (31).

In the present study, the gene expression profiles of MCF 7 cells treated with 3 and 10 μmol/l genistein were analyzed, respectively. The results showed that the number of DEGs in the cell cycle was increased in the 10 μmol/l genistein group as compared with the 3 μmol/l genistein group, and the function of cell proliferation was enriched in the 10 μmol/l genistein group. This suggested that a high concentration of genistein could initiate more marked changes in the expression of the DEGs. The most significant function of the DEGs in the overlapping network was the cell cycle, involving 47 DEGs, including CDC20, BUB1, MCM2 and cyclin B1. These genes were also involved in the cell cycle pathway, which was the most significant pathway in the pathway enrichment analysis. CDC20 is an essential cell-cycle regulator required for the completion of mitosis. CDC20 binds to and activates the ubiquitin ligase activity of the anaphase-promoting complex/cyclosome (APC/C), and enables the ubiquitination and degradation of securin and cyclin B, thus promoting the onset of anaphase and completion of mitotis (32). The mRNA and protein levels of CDC20 and BUB1 have been shown to be significantly higher in breast cancer cell lines and in high-grade primary breast cancer tissues. In addition, the upregulation of BUB1 protein is used as a marker, as it is upregulated in ~80% of breast cancers in paraffin-embedded tissues (33). Upregulation of cyclin B1 has been associated with poor prognosis in hormone receptor-positive, luminal B and basal-like breast cancers (34). MCM-2 has been reported for its use as a strongly independent prognostic marker in breast cancer and non-small cell lung cancer (35,36), in addition to the standard proliferation marker Ki-67. MCM2 and BUB1 have additionally been identified to be involved in cell cycle progression (37). Therefore, the cell cycle may be important role in the development of breast cancer. In this study, the expression levels of CDC20, BUB1, MCM2, and cyclin B1 were upregulated in the 3 and 10 μmol/l genistein groups, indicating the promoting effects of genistein on cancer cell proliferation. However, inhibition effects of genistein on cancer cell proliferation also exist and act via the cell cycle.

Pathway enrichment analysis further confirmed the participation of these DEGs in the cell cycle. Cell cycle arrest caused by genistein occurs during different phases of the cell cycle, including G_2_/M, G_0_/G_1_ and G_1_/S phase. In a previous study, Cappelletti *et al* (38) demonstrated that genistein could restrain breast cancer cells to the G_2_/M phase (38). The accumulation of genistein-treated cells have additionally been shown to exist in the S and G_2_/M phases of the cell cycle, and undergo apoptosis (39). Genistein could induce the up- and downregulation of apoptosis-associated genes, including Bax-2, p21WAF1, Bcl-2 and p53 (40), and the ratio of Bax and Bcl-2 were previously demonstrated to be important for the survival of cells (41). Therefore, genistein could inhibit the cell cycle in breast cancer, resulting in cellular apoptosis. Notably, the GLIPR1 gene was downregulated in the 3 μmol/l genistein group, while upregulated in the 10 μmol/l genistein group. GLIPR1, also termed RTVP1, encodes glioma pathogenesis-related protein 1, which has p53-regulated proapoptotic activities, and is downregulated in prostate and bladder cancer cells (42). The discrepancy in the GLIPR1 expression between the two genistein groups indicated that the effects of genistein are dose-dependent, and genestien only inhibits cancer at a high concentration.

In conclusion, the cell cycle may be an important pathway based on the analysis of MCF-7 breast cancer cells treated with 3 and 10 μmol/l genistein, respectively. This revealed that the cell cycle may be an important pathway in the mechanisms underlying the treatment of breast cancer with genistein. The identified DEGs, which were involved in cell cycle, including CDC20, BUB1, GLIPR1, MCM2, and CCNB1, could have a crucial function in the development of breast cancer, and may become potential targets or prognostic markers for breast cancer. Experimental verification is required in future studies.

## Figures and Tables

**Table I:** Result of functional enrichment of differentially expressed genes in the overlapping network.

Term	Count	FDR
GO:0007049~ cell cycle	47	4.02×10^−31^
GO:0000280~ nuclear division	30	1.39×10^−27^
GO:0007067~ mitosis	30	1.39×10^−27^
GO:0000087~ M phase of mitotic cell cycle	30	2.39×10^−27^
GO:0048285~ organelle fission	30	4.64×10^−27^
GO:0000279~ M phase	33	1.43×10^−26^
GO:0006260~ DNA replication	28	2.51×10^−26^
GO:0022403~ cell cycle phase	35	4.92×10^−26^
GO:0000278~ mitotic cell cycle	33	6.18×10^−25^
GO:0022402~ cell cycle process	36	9.46×10^−23^
GO:0051301~ cell division	29	2.00×10^−22^
GO:0006259~ DNA metabolic process	33	1.14×10^−20^
GO:0051726~ regulation of cell cycle	25	5.84×10^−16^
GO:0006974~ response to DNA damage	20	2.95×10^−9^
GO:0033554~ cellular response to stress	22	7.08×10^−8^

FDR, false discovery rate.

**Table II:** Result of pathway enrichment of differentially expressed genes in the overlapping network.

Term	Count	FDR
hsa04110:Cell cycle	20	1.52×10^−16^
hsa03030:DNA replication	9	5.95×10^−7^
hsa04114:Oocyte meiosis	11	3.16×10^−5^

FDR, false discovery rate.
